# Study of exopolysaccharide produced by *Streptomyces rochie* strain OF1 and its effect as ameliorative on osteoarthritis in rats via inhibiting TNF-α/COX2 pathway

**DOI:** 10.1186/s43141-023-00471-3

**Published:** 2023-02-09

**Authors:** Sahar Saleh Mohamed, Mohamed E. El Awady, Sayeda Abdelrazek Abdelhamid, Ahmed Abdelghani Hamed, Abeer A. A. Salama, Manal S. Selim

**Affiliations:** 1 Microbial Biotechnology Department, National Research Centre, Giza, Egypt; 2 Microbial Chemistry Department, National Research Centre, Giza, Egypt; 3 Pharmacology Department, National Research Centre, Giza, Egypt

**Keywords:** Exopolysaccharide, *Streptomyces rochie*, Antimicrobial, Antibiofilm, Inflammatory biomarkers

## Abstract

**Background:**

Carbohydrates are known as the main natural products of life activities.

**Results:**

*Streptomyces rochie* strain OF1 isolated from a mangrove tree produced exopolysaccharide S5 (EPSS5) (14.2 gl^−1^) containing uronic acid 21.98% sulfate content of 11.65 mg/ml, and a viscosity of 1.35 mm^2^/s. while total hexose amine content was 24.72%. The high performance liquid chromatography (HPLC) analysis of mono sugars revealed that EPS was composed of manouronic acid, glucuronic acid, xylose, and fructose at a molar ratio of 1.0:0.5:1.0:2.0, respectively. It showed that the whole antioxidant activity was 92.06%. It showed antibacterial activity against *Staphylococcus aureus*, and *E. coli*, MRSA and *Klebsiella pneumoniae*. But, EPSS5 displayed low antifungal activity against *Candida albicans*. While no antifungal activity has been detected against *Aspergillus niger*. EPSS5 has antibiofilm action that is noticeable toward *S. aureus* with an inhibition ratio of biofilm up to 50%. Effect of EPS on serum levels of TNF-α and COX2 by 2 fold and 1.9 fold of EPS reduced serum levels of Tumor necrosis factor-α (TNF-α) by 38%, 12%, 49%, and Cyclooxygenase-2 (COX2) by 61%, 34%, and 62%, respectively. By affected of EPSS5 on arthritis in rats stimulated by carrageenan.

**Conclusions:**

Administration of EPS ameliorated carrageen-induced elevation in inflammatory mediators; TNF-α/COX and suppressed the expressions of metalloproteinase 9 (MMP9) by 68%, 86%, and 75% correspondingly in comparison to the group of carrageenans. Then again, therapy involving a high dose only reduced MMP9 level by 57%, compared to free drug suggesting that EPSS5 is a good inhibitor of the MMP9, as it brought MMP9 back to normal levels via the signaling pathway.

## Background

A few years ago, there has been a growing awareness of the medical benefits of secondary metabolites extracted from marine microorganisms by microbial fermentation [1, 2]. Carbohydrates, along with proteins, lipids, and nucleic acids, are commonly found in biological organisms and are known as the main natural products of life activities [3]. A polysaccharide (PS) is a carbohydrate that was primarily produced by bacteria, fungi, microalgae, and streptomycetes. That had two forms an attachment shape to the cell wall called a capsular polysaccharide (CPS) or a free form known as exopolysaccharide that was released into culture (EPS) [4–6]. Sulfate polysaccharide is a form of polysaccharide with sulfate groups on the hydroxyl group [3, 7]. Microbes that synthesized polymeric compounds as polysaccharides may be found in the marine environment [8, 9]. Indeed, EPSs that were produced by marine microorganisms as extreme habitats showed variety of biotechnological industries [10, 11]. The exopolysaccharides had strong biological activities and played roles in the regulation of cell division and differentiation, immune regulation, and also antioxidant, antitumor, and antiviral activities, preventing inflammation and atherosclerosis [12–14]. Osteoarthritis (OA) is a chronic and destructive disease marked by articular cartilage degeneration and the production of osteophytes [3]. Joint inflammation, swelling, deformities, and restricted mobility were some of the clinical symptoms [15]. Age, oxidative stress, excessive weight-bearing, physiology, and biomechanical environment changes in joints were all causes that could also lead to OA [16]. Therefore, in this study, we would be stimulated to investigate bioactive polysaccharides (EPS) produced by marine *Streptomyces* sp. to inhibit the TNF-α/COX2 pathway in the treatment of osteoarthritis and study its effect as an antimicrobial, antibiofilm, and antioxidant.

## Methods

### Collection of samples and isolation of streptomycetes

Marine sediment sample collected from mangrove tree around the rhizosphere (Red Sea governorate, Red Sea, Egypt) was suspended in 95 ml of sterile water. Serial dilutions of sediment samples were plated on marine starch nitrate plates according to [17] using 75% sea water and incubated at 28 °C for 7–14 days to allow the slow growing forms to develop. Streptomycetes were isolated based on their specific morphological characteristics and then subjected to purification.

### Screening for best strain produced EPS

A loop was full of the strain G4 was inoculated into a 250-ml conical flask containing 50 ml of broth medium containing (g/l) Glucose 10.0, Tryptone 5.0, Yeast extract 5.0, K_2_HPO_4_ 3.0, NaCl 3.0, KH_2_PO_4_ 1.0, MgSO_4_.7H_2_O 0.5, CaCO_3_ 0.5 which dissolved in 750 ml sea water then completed to 1L with 250 ml distilled water, pH 7 [18] and incubated on a shaker at 150 rpm for 7 days at 28 °C. The cell-free culture supernatant was obtained by centrifugation at 5000 rpm for 20 min; the supernatant was mixed with trichloroacetic acid (TCA) (10%) and keeping it overnight at 4 °C under static conditions. Then, centrifuged at 5000 rpm for 20 min to remove protein and the pH of supernatant was neutralized to 7 with NaOH solution [19]. The EPSs containing solution was completed to 4 volumes with cold absolute ethanol and kept at 4 °C overnight for allowing precipitation of EPS. The precipitated EPSs were separated by centrifugation at 5000 rpm for 20 min and re-suspended in deionized water then washed twice, and re-precipitated by 4 volume of cold absolute alcohol and dried at 50 °C. For fractionation and major fraction determination, absolute cold ethanol was added 1, 2, 3, and 4 volumes gradually and the precipitated EPS was collected up to date. The major fraction which obtained by two volume was dialyzed three times (1000 ml × 3) against flowing tab water using dialysis tubing (MWCO 2000) for 72 h, washed twice with acetone, dehydrated by ether, dried at 40 °C. The deproteinated solution through precipitation with 3 volume chilled absolute ethanol, which was later collected by centrifugation at 4 °C at 5000 rpm for 20 min and dissolved in deionized water. The solution was lyophilized to obtain the dry exopolysaccharide. EPS production was determined by quantifying the carbohydrate content of the pellets as glucose equivalents using the phenol-sulfuric acid method [20].

### Identification of the promising streptomycete strain

#### Morphological, physiological, and biochemical characterization

The promising streptomycete strain that have high EPS productivity (S5) was subjected to morphological, physiological and biochemical identification. The spore chain morphology and the number of spores per chain of the strains of 14-day-old cultures grown on inorganic salts-starch agar were examined by light microscope [21]. The spore surface was examined using Em10 Carl-Zeiss transmission electron microscope [22]. The cultural characteristics of the strains were tested on the basis of the methods used in the International Streptomyces Project (ISP), using the media recommended by Shirling and Gottlieb [21]. The colors of mature sporulating aerial mycelium and substrate mycelium were monitored for 7, 14, and 21-day-old cultures grown on starch nitrate medium. Diffusible pigments were detected on glycerol asparagine agar medium. Color determination was carried out using ISCC-NBS color charts [23]. Physiological and biochemical characterizations were determined according to the methods given by several authors as follows: (i) melanin pigment production [24], (ii) nitrate reduction [25], (iii) pectinase [26], and (iv) hydrogen sulphide production [27].

### Molecular identification

The most promising streptomycete strain (S5) was identified by 16S rRNA gene sequencing. Chromosomal DNA was extracted using the DNeasy Power Soil Kit (Qiagen) according to the manufacturer’s instruction. 16S rRNA gene was amplified using universal primers F (5′-GTGCCAGCAGCCGCGGTA-3′) and R (5′-TTGTAGCACGTGTGTAGCCC-3′) (according to Kröger, M., Institute of Microbiology and Molecular Biology, University of Gießen, Germany). PCR reaction was achieved in a volume of 50 ll containing 1× green Taq PCR Buffer, 200 mM of each dNTPs, 100 mg BSA, 10 pmole of each primer, 2.5 U of Taq DNA polymerase (Sigma) and 10 ng of DNA. PCR was achieved by the following conditions: 1 min at 98 °C followed by 35 cycles of 1 min at 94 ^o^C, 30 s at55 ^o^C, and 1 min at 72 ^o^C. The PCR product was purified using the QIAquick PCR Purification Kit (Qiagen) and sequenced in Lab Technology Company (Cairo, Egypt). The 16S rRNA sequence was matched with previously published 16S rRNA sequences of bacteria in the National Center for Biotechnology Information (http://www.ncbi.nlm.nih.gov) [28], using BLAST, moreover at the EzBioCloud Server [29]. Selected sequences of other microorganisms with the greatest similarity to the 16S rRNA sequences of the bacterial strain were extracted from the nucleotide sequence databases and aligned generating phylogenetic tree. The 16S rRNA gene sequence of the bacterial isolate was deposited in the GenBank nucleotide sequence database with accession number ON386190.

### Characterization of exopolysaccharide

#### Fourier-transform infrared spectroscopy (FTIR)

The Fourier-transform infrared (FTIR) spectrum of the exopolysaccharide was recorded on a Bucker scientific 500-IR Spectrophotometer. The sample was mixed with KBr powder, ground, and pressed into 1 mm pellets for FTIR measurements in the range of 400–4000 cm^–1^ [30].

### Chemical analysis for EPS

Sugar identification done by comparison with authentic sugars. The uronic acid content of the EPS was determined by the *m*-hydroxybiphenyl method [31, 32]. Sulfate was determined using the turbid method [33]. Determination of total hexose amine content done according to Satake et al*.* [34]. The monosaccharides contents were quantified by HPLC on a Shimadzu Shim-Pack SCR-101N column (7.9 mm × 30 cm), using deionized water as the mobile phase (flow rate 0.5 ml/min), as described by Kwon and Kim [35].

### Molecular weight determination

The molecular weight of ESP was determined according to Jun et al. [36]. Homogeneity and average molecular weight (Mw) of EPS.

### Viscosity of sulfate content in exopolysaccharide

The kinematics exopolysaccharide (2 mg/ml) was measured at 40 °C by Technico viscometer (BS/U 415). The time required for the level of the liquid to drop from one mark to the other was measured with a stopwatch. The average of not fewer than three readings give the flow time of the EPS sample to [37].

### Antioxidant determination of EPS

The free radical-scavenging activity of exopolysaccharide was measured by 1,1-diphenyl-2 picrylhydrazyl (DPPH) radicals using the method of Shimada et al. [38].

### Antimicrobial activity of EPS

To measure the antibacterial activity of the crude and pure compounds. Gram-positive bacteria (*Staphylococcus aureus* NRRLB-767 and MRSA), Gram-negative bacteria (*Escherichia coli* ATCC 25922, *Klebsiella pneumoniae* ATCC 10145), yeast (*Candida albicans* ATCC 10231) and fungi (*Aspergillus niger* NRRLA-326) were used as test organisms and antibacterial tests were performed. The tests were performed in 96-well flat polystyrene plates. Ten microliters of test extracts (final concentration of 500 μg/ml) were added to 80 μl of lysogeny broth (LB broth) followed by addition of 10 μl of bacterial culture suspension (log phase), then the plates were incubated overnight at 37 °C. After incubation, the positive antibacterial effect of the tested compounds observed as clearance in the wells, while compounds that did not have an effect on the bacteria, the growth media appeared opaque in wells, the control is the pathogen without any treatment. The absorbance was measured after about 20 h at OD600 in a Spectrostar Nano Microplate Reader (BMG LABTECH GmbH, Allmendgrun, Germany)

### In vitro antibiofilm activity of EPS

The microtiter plate assay (MTP) method was used to measure the biofilm inhibitory activity of the isolated compound in 96 well-flat bottom polystyrene titer plates and four clinical microbes (*E. coli* and *S. aureus*) according to Shalabi and Eskander, [39]. Each well of the 96 well-plate was filled with 180 μl of lysogeny broth (LB), with the following composition (g/l): Tryptone, 10.0; yeast extract, 5.0; NaCl, 10.0. At pH 7.2, 10 μl of overnight growing test bacteria, 10 μl of the isolated pure compound at a concentration of 100 μg ml^−1^ sample, along with the negative control (i.e., filtrate without sample) and then the plate was incubated for 24 h at 37 °C. The content of each well was removed, and each well was washed with 200 μl of phosphate buffer saline pH 7.2 to eliminate the floating bacteria. Crystal violet (0.1%, w/v) was added to each well for 1 h, for staining, then 200 μl of distilled water was used, for washing, and then the plate was kept for drying in the laminar flow. For measuring the optical density (OD) at 570 nm, 95% ethanol was added to the dried plate, and by using (SPECTRO star nano absorbance plate reader–BMG LABTECH).

### Effect of EPS on inflammatory biomarkers TNF-α, COX2, and MMP9

#### Animal

Wister albino male rats of 140–150 g were provided by the Animal House of the National Research Centre (Cairo, Egypt). The rats were group-housed under temperature and light-controlled conditions (24 ± 2 °C under a 12 h light/dark cycle) and had free access to standard laboratory rodent chow and water. The animal experiments were performed by the guidelines of the Institutional Animal Ethics Committee (Medical Research Ethics Committee (MREC) of the NRC, Cairo, Egypt. Experimental protocol was approved (Number: 16-438).

#### Chemicals

Carrageenan was purchased from Sigma Aldrich. Tumor necrosis factor-α (TNF-α) was purchased from Elabscience, China. Cyclooxygenase-2 (COX-2) and Metaloprotenase 9 (MMP9) were obtained from NOVA, Beijing, China.

### Experimental design of the in vivo study

The induction of arthritis was achieved by intra-articular injecting of carrageenan (0.1 ml/joint) into a rat's right knee for 10 days [40]. Rats were divided randomly into five groups each group comprises 8 male rats as follows: group 1: normal control. Group 2: positive control (carageenan) group. Group 3: rats treated orally with ref drug glucosamine (300 mg/kg). Groups 4 and 5: rats treated orally with EPS (50 and 100 mg/kg) for 10 days, concurrent with carrageenan.

### Blood sample preparation

At the end of the experimental period, blood samples were collected from the retro-orbital venous plexus by heparinized capillary tubes under anesthesia. Blood samples were centrifuged at 3000 rpm for 10 min. The sera were stored at – 20 °C until examined for determination of biochemical parameters [41].

### Effect of EPS on inflammatory biomarkers TNF-α, COX2, and MMP9

TNF-α, COX2, and MMP9 serum levels were assessed by Elabscience, China, and NOVA, Beijing, China ELISA kit. The manufacturer's instructions for the kit were followed, for estimating the results. Samples and standards were pipetted into the wells with immobilized antibodies specific for rat TNF-α, COX2, and MMP9 and then were incubated. After incubation and washing, biotinylated antirat antibodies were added. Any unbound substances were washed away, and horseradish peroxidase-conjugated streptavidin was pipetted into the wells, which were washed once again. TMB (tetramethylbenzidine) substrate solution was added to the wells; color developed proportionally to TNF-α, COX2, and MMP9 bound amount. Color development was discontinued (stop solution) and the color intensity was measured at 450 nm.

### Statistics

All the values are presented as means ± standard error of the means (SE). Comparisons between different groups were carried out using one-way analysis of variance (ANOVA) followed by Fisher’s LSD test for multiple comparisons. GraphPad Prism software, version 5 (Inc., USA) was used to carry out these statistical tests. The difference was considered significant when *p <* 0.05.

## Results

### Production of EPS and identification of the promising isolate

**Fig. 1:**
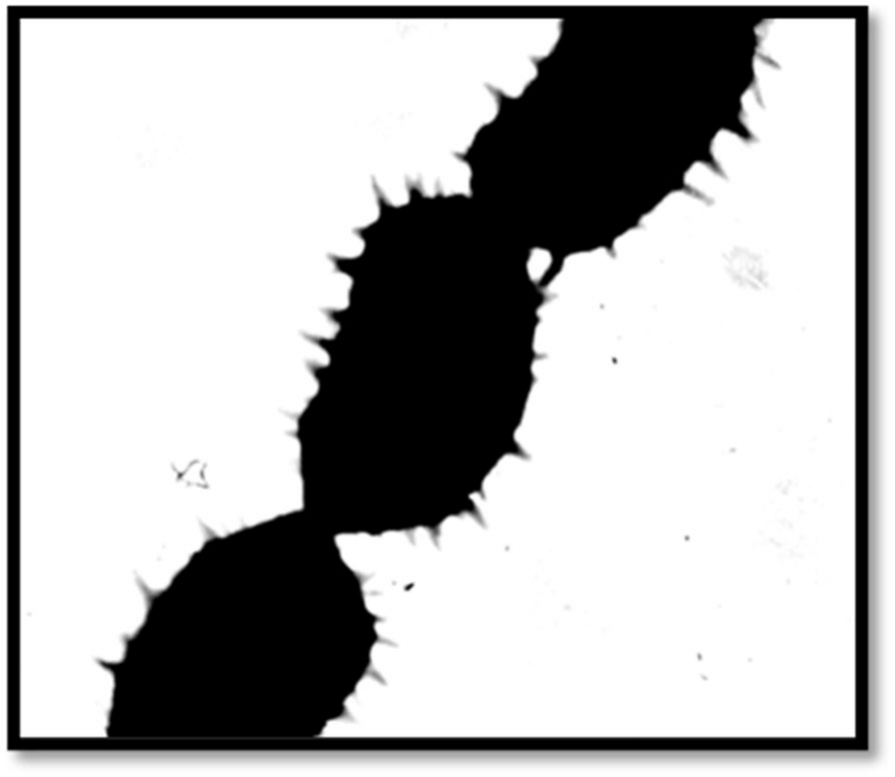
Electron micrograph of spores of *Streptomyces rochie* strain OF1 (X 14 000)

**Fig. 2:**
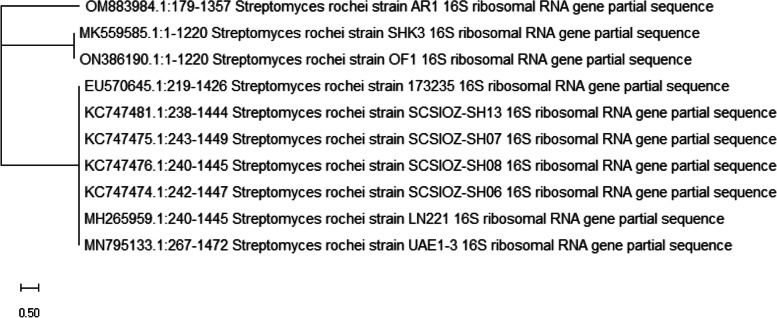
Phylogenetic tree of *Streptomyces rochie* strain OF1, a local isolate in a using its incomplete 16S rRNA sequencing in comparison to nearby sequences found in GenBank databases

Seven streptomycetes isolates (S1–S7) were isolated from marine sediment sample around the rhizosphere of mangrove tree (Red Sea, Egypt) using the morphology of the colony. Therefore, the amount of carbohydrates in their supernatant broth was determined. Exopolysaccharides could be produced by the majority of the isolates, but (S5) was selected for further study owing to its greatest EPS manufacturing capacity (14.2 g/l) and the significant fraction was achieved by adding two volumes 100% ethanol and also known as EPSS5. Considering its physiological, morphological, and also the biochemical characteristic as displayed in (Table 1). The isolate’s spore mass was grey in appearance with no melanoid and diffusible pigments. Additionally, it demonstrated a variation in how various sugars were used as a carbon source. In contrast, the spore chains were spiral and had spiny surfaces (Fig. 1). The isolate had 100% of *Streptomyces rochie*, according to analysis of the 16S rRNA gene sequencing of the promising strain (S5) with information from GenBank. So, the strain was identified as *Streptomyces rochie strain OF1 with accession number ON386190* as shown in phylogenetic tree (Fig. 2).

**Table 1:** The Morphology, physiology and biochemistry of the *Streptomyces rochie* strain OF1

Morphological and cultural characteristics								
Spore chain morphology		Spore surface ornamentation	Color of spore mass	Pigmentation of substrate mycelium		Diffusible pigment		
Open spiral > 40		spiny	grey	yellow		−ve		
Physiological and biochemical characteristics								
Melanin pigment production		Degradation activities			Chitin decomposition	Pectin decomposition	Nitrate reduction	H_2_S production
Peptone iron	Tyrosine	Xanthine	Elastin	Arbutin	−ve	−ve	−ve	+ve
−ve	−ve	+ve	−ve	+ve				
Utilization of sugars								
D-fructose	Sucrose	Rhamnose	D-mannitol	D-xylose	Raffinose	I-inositol	Galactose	L-arabinose
−ve	+ve	+ve	+ve	+ve	+ve	+ve	+ve	+ve

### Characterization of EPS

**Fig. 3:**
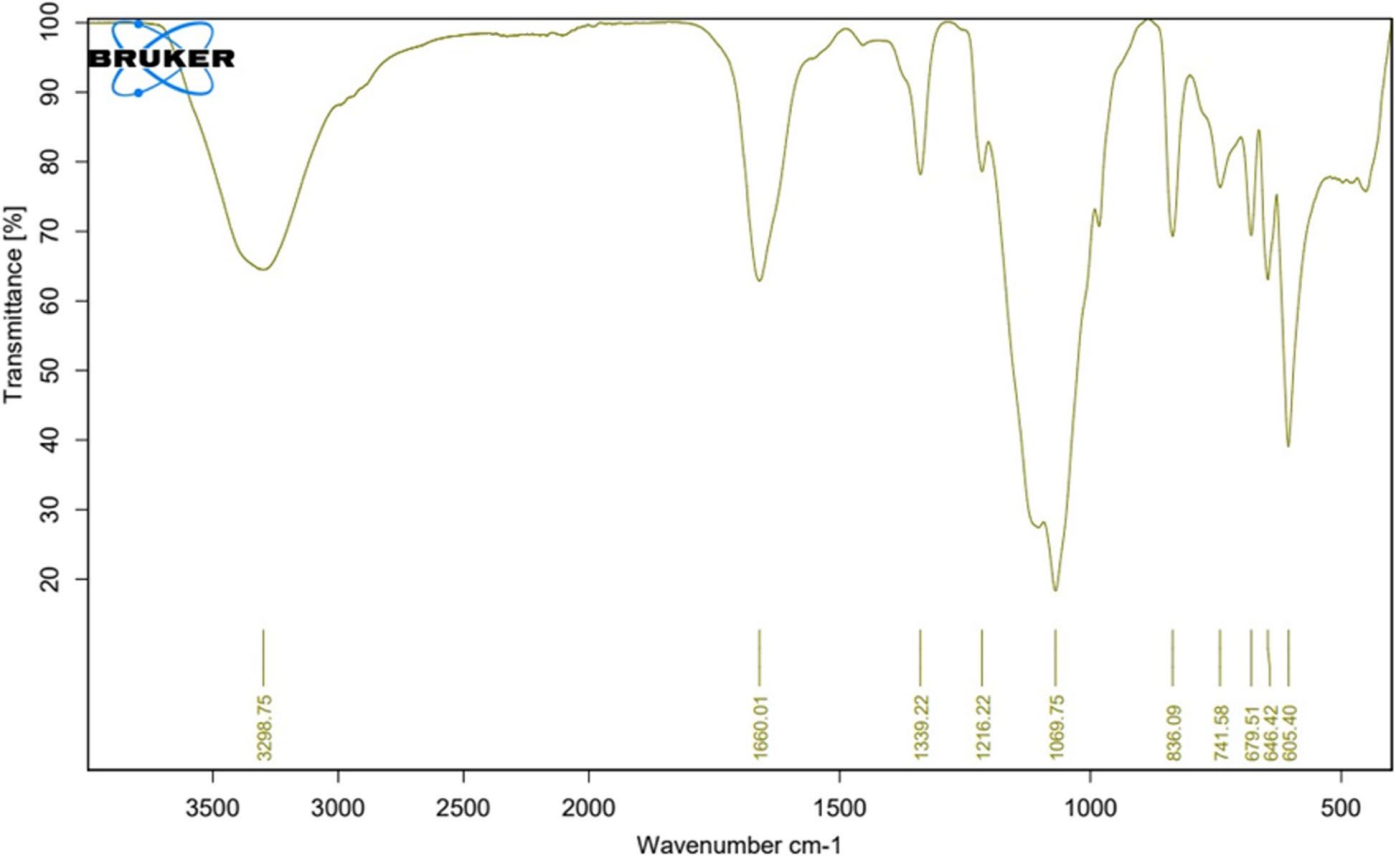
FTIR for *Streptomyces rochie* strain *OF1* EPS

EPSS5 produced by *Streptomyces rochie* showed the uronic acid was 21.98% indicating that the produced EPS was acidic while the sulfate content was 11.65 mg/ml, and the viscosity 1.35 mm^2^/s. while, total hexose amine content was 24.72%. The HPLC analysis of mono sugars revealed that EPS was composed of manouronic acid, glucuronic acid, xylose, and fructose at a mole ratio of 1.0:0.5:1.0:2.0, respectively. Infrared spectra of the polysaccharide content shown in Fig. 3, had a broad extending powerful distinctive peak at approximately 3298.75 cm^−1^ because of polysaccharide hydroxyl stretching vibration. As well as, Signals at 1660.01 cm^−1^ represented COO^−^ stretch vibration and 1339.22 cm^−1^ (symmetrical COO^-^ stretching vibration), proving the existence of uronic acid. The absorptions around 1069.75 cm^−1^ indicated the SO^=3^ and the characteristic absorptions at 836.09 cm^−1^ in the IR spectra indicated that α-configurations were simultaneously present in ESP.

### Evaluation of antioxidant activity of EPSS5

**Fig. 4:**
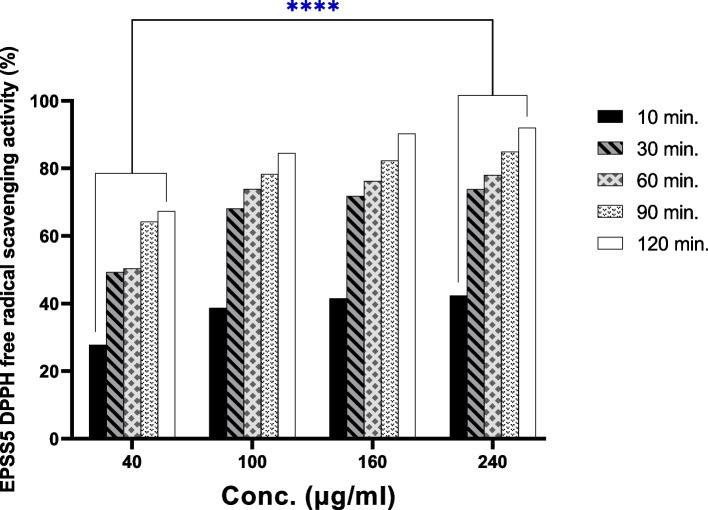
EPSS5 free radical scavenging capability against DPPH

The EPSS5's DPPH radical-scavenging activity as shown in Fig. 4, from which it was obvious that the EPS has a scavenging efficiency of an IC_50_ value of 40 μg/ml after 60 min. The exopolysaccharide showed high powerful free radical scavenging activity (92.06%) after 120 min.

### Antimicrobial activity of EPSS5

**Fig. 5:**
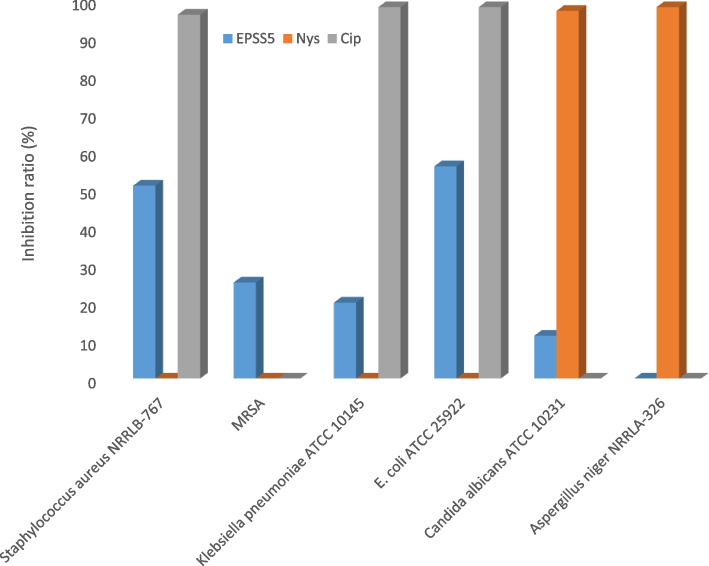
Antimicrobial activity of EPSS5

The antimicrobial activity of the EPSS5 against pathogenic bacteria and fungi was carried out using 96-well flat polystyrene plates Fig. 5. The obtained results revealed that, EPSS5 displayed a moderate antibacterial activity against *E. coli* and *Staphylococcus aureus*, while it showed low antibacterial activity against MRSA and *Klebsiella pneumoniae*. On the other hand, EPSS5 displayed low antifungal activity against *Candida albicans*. While no antifungal activity has been detected against *Aspergillus niger*. The MIC of the EPSS5 was measured and presented in (Table 2).

**Table 2:** Minimum inhibitory concentration of EPSS5

	MIC (μg/ml)				
	*Staphylococcus aureus* NRRLB-767	MRSA	*Klebsiella pneumoniae* ATCC 10145	*E. coli* ATCC 25922	*Candida albicans* ATCC 10231
EPSS5	12.50	50.00	25.00	12.50	50.00
Nys	–	–	–	–	0.390
Cip	0.50	–	0.750	0.390	–

### Antibiofilm activity of EPSS5

**Fig. 6:**
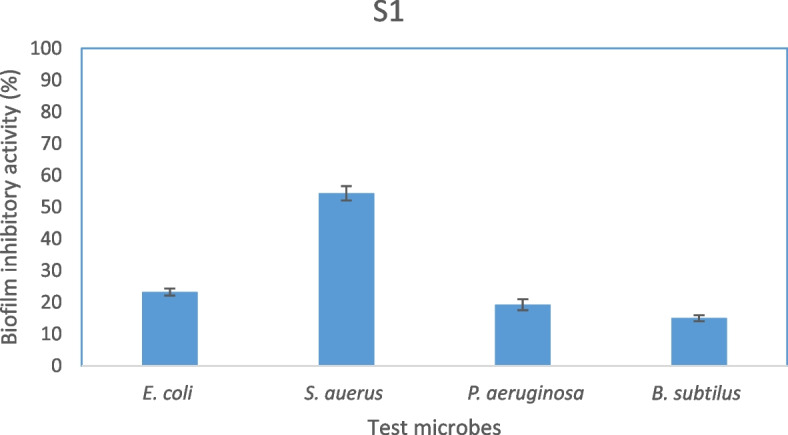
Biofilm inhibitory activity of EPSS5

The antibiofilm activity of the EPSS5 was measured using MTT assay towards four pathogenic bacteria includes (*S. aureus*, *P. aeruginosa*, *E. coli*, and *B. subtilis*). The obtained results showed that, EPSS5 has clearly detectable antibiofilm action toward *S. aureus* with a 50% biofilm inhibition ratio, while weak biofilm inhibition activity against *P. aeruginosa, E. coli*, and *B. subtilis* (Fig. 6).

### Effect of EPSS5 on TNF- and COX2 levels in serum

**Fig. 7:**
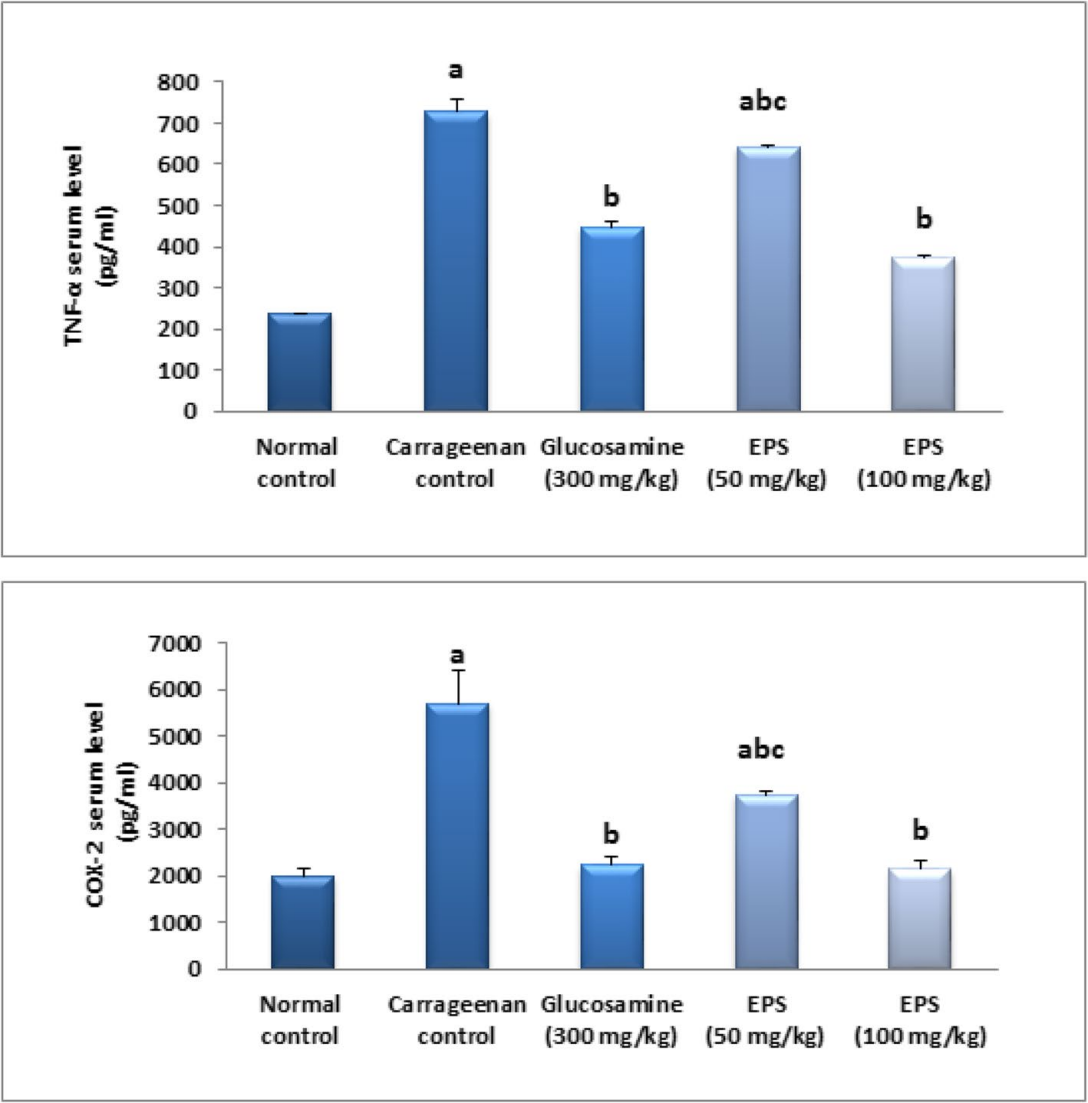
Effect of EPSS5 on serum levels of TNF-α and COX2. The data was presented as mean ± SE (*n* = 8). Fisher‘s LSD test for multiple comparisons was used after one-way ANOVA in the statistical analysis. At *P* ˂ 0.05, various letters show significant differences

The findings showed that TNF- and COX2 serum levels were raised in arthritis induced group by 2-fold and 1.9-fold, respectively, in comparison to the norm values. Therapy using the ref, both doses of EPSS5 reduced serum levels of TNF-α by 38%, 12%, and 49% and COX2 by 61%, 34%, and 62%, correspondingly in comparison to the carrageenan group. In addition, therapy using a high dose returned TNF-α and COX2 serum levels to their normal value (Fig. 7).

### Effect of EPSS5 on the MMP9 signaling pathway

**Fig. 8:**
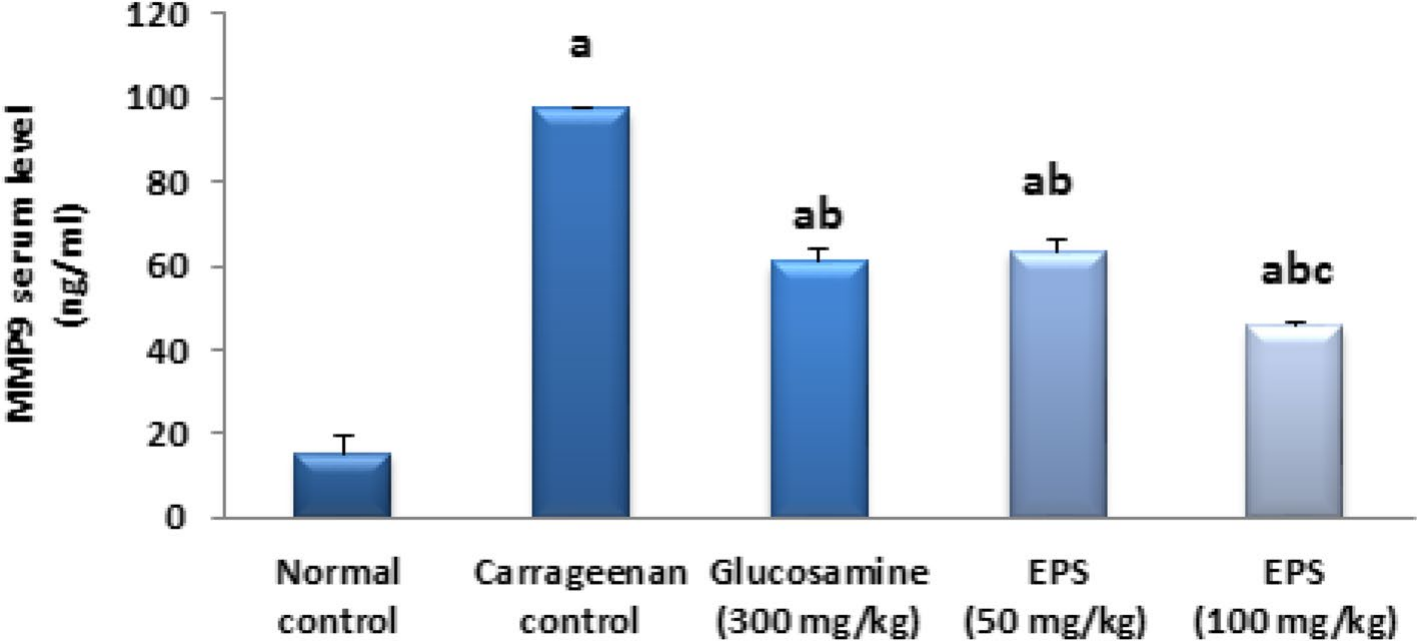
Effect EBS on MMP9 serum level. The data were presented as mean ± SE (*n* = 8). Fisher's LSD test for multiple comparisons was used after one-way ANOVA in the statistical analysis. At *P* ˂ 0.05, various letters are statistically distinct

In the current study, MMP9 serum level was raised in the carrageenan group by 5-fold, in comparison to the norm. Treatment with the ref, both doses of EPSS5 reduced serum levels of MMP9 by 68%, 86%, and 75% respectively as compared to the carrageenan group. In addition, treatment with a high dose reduced just MMP9 level by 57%, compared to free drug suggesting that F1 is a good inhibitor of the MMP9 signaling pathway as it brought MMP9 back to its typical values (Fig. 8).

## Discussion

Mangrove ecosystem is highly productive ecosystems with soil conditions- high salinity, high moisture, high organic matter content, and low oxygen- extremely different from normal soil conditions; therefore, mangrove actinomycetes produce unique bioactive compounds [18, 42]. About 86 new species of actinobacteria (including 8 novel genera) have been isolated from mangrove environment. Mangrove actinobacteria have produced 84 new compounds with different biological activities (antimicrobial, antitumor, antiviral, antifibrotic, and antioxidants) including salinosporamides, xiamycins, and novel indolocarbazoles [43]. Seven streptomycetes isolates were isolated on marine starch nitrate medium from mangrove trees around the rhizosphere. Screening of the production of the exopolysaccharide on a specific medium the highest produced isolate was selected. Our finding was similar to another study that reported that was first isolated from Egyptian marine [10]. Based on its characteristic and molecular identification, the best isolate was identified as Streptomyces rochie strain OF1 that similar study by Abdallah et al. [12] they were identified the promising streptomycete isolate that produced EPS as Streptomyces xiamenensis. Components most commonly found in marine EPS were monosaccharides including pentoses (xylose, ribose, arabinose), hexoses (glucose, mannose, galactose, rhamnose, allose, fucose), Galactosamine and glucosamine, two amino or glucuronic acids, galacturonic acids, two uronic acids [44]. In our study, the mono sugars revealed that EPS was composed of manouronic acid, glucuronic acid, xylose, and fructose at a molar ratio of 1.0: 0.5: 1.0: 2.0, respectively. The EPS with uronic and sulfate groups increased its activity as an anti-inflammatory antioxidant, and an antitumor agent [12]. EPS produced by *Streptomyces rochie* strain OF1 showed the uronic acid was 21.98 % and the sulfate content was 11.65 mg/ml, and the viscosity was 1.35 mm^2^/s indicating that antioxidant was high efficiency when using the DPPH radical-scavenging of this EPS. When bacteria, viruses, fungi, and parasites change over time and lose their capacity to respond to antibiotics, antimicrobial resistance (AMR) occurs, making infections harder to cure and increasing the risk of disease transmission, life-threatening illness, and death. Antimicrobial drugs, including antibiotics become ineffective because of medication resistance, and illnesses become less likely to succeed or be possible to treat [12]. EPSS5 showed moderate antibacterial activity against *Staphylococcus aureus*, and *E. coli* and showed low antifungal activity against *Candida albicans*. While, no antifungal activity has been detected against *Aspergillus niger*. Bacterial biofilms are intricate populations of bacteria adhered to surfaces and held together by self-produced polymer matrixes, which mostly consist of secreted proteins, extracellular DNAs, and polysaccharides [5]. Bacterial biofilms have been found to contribute significantly to bacterial persistence and are a source of nosocomial infection that are engaged in a variety of infectious illnesses [45]. So, the ant biofilm activity of the EPSS5 was measured aganist four pathogenic bacteria including (*P. aeruginosa, S. aureus, E. coli*, and *B. subtilis*). EPSS5 has antibiofilm activity toward *S. aureus* with biofilm inhibition. While, weak biofilm inhibition activity against *P. aeruginosa*, *E. coli*, and *B. subtilis.* Osteoarthritis was a pervasive joint inflammatory disease and a cause of suffering pain and disability [46] that was characterized by synovial membrane inflammatory reaction [47], that causes cartilage damage in the joints [48]. Fibroblast-like synoviocyte (FLS) was an important cell that was attributed to arthritis [49]. These cells when activated, secret pro-inflammatory cytokines and chemokines that promote joint destruction through the release of matrix metalloproteinases (MMPs) [50]. TNF Inflammatory cytokine produced by FLS contributes to osteoclast stimulation [51, 52] leading to cartilage damage [53, 54]. Injecting carrageenan intra-articularly induces inflammatory mediators released such as TNF-α, COX2, and prostaglandin E2 (PGE2) resulting in inflammation in the knee joint [55]. A membrane-bound, heme-containing glycoprotein is called COX. There are two COX isoforms: COX-1 and COX-2, both of which have a similar structure and catalytic activity. Despite the fact that it was clear that COX-2 was the key enzyme controlling PGE2 synthesis through the arachidonic acid route in the inflammation process. The expression of COX-2 and TNF- was clearly reduced by EPS [56] states that Sulfated Polysaccharide from *Sargassum swartzii* was found to be working by inhibiting inflammatory mediators such as also reduce the levels of COX-2 and TNF-α.

## Conclusion

In this study, EPSS5 was a major fraction of EPS produced from the locally Egyptian isolate *Streptomyces rochie* stain OF1. EPSS5 have strong biological activity as antioxidant, antimicrobial, anti-biofilm and exhibits good effects on rheumatoid arthritis (RA). Ameliorated the underlying inflammatory response of carrageenan-induced arthritis, which enabled it to be a therapeutic drug for treating arthritis via regulating TNF-α/COX2/MMP9. This research focus on analyzing the chemical composition of EPSS5 to explain the biological activity in relation to the metabolites produced by this strain.

## Data Availability

The datasets generated during and/or analyzed during the current study are available from the corresponding author on reasonable request.
